# Supplementary material for: Altered microRNA Transcriptome in Cultured Human Liver Cells upon Infection with Ebola Virus
Source: Int J Mol Sci. 2021 Apr 6;22(7):3792. doi: 10.3390/ijms22073792 (PMC8038836; doi:10.3390/ijms22073792)
Supplement: Supplementary file 1 [file ijms-22-03792-s001.zip › Supplementary_File/C_ GO_Analysis_Results/16-30nt_go_Mayinga-96h-Huh7_vs_Control-96h-Huh7_down.mature_mirna_targets/CC_Pvalue_tree.pdf]

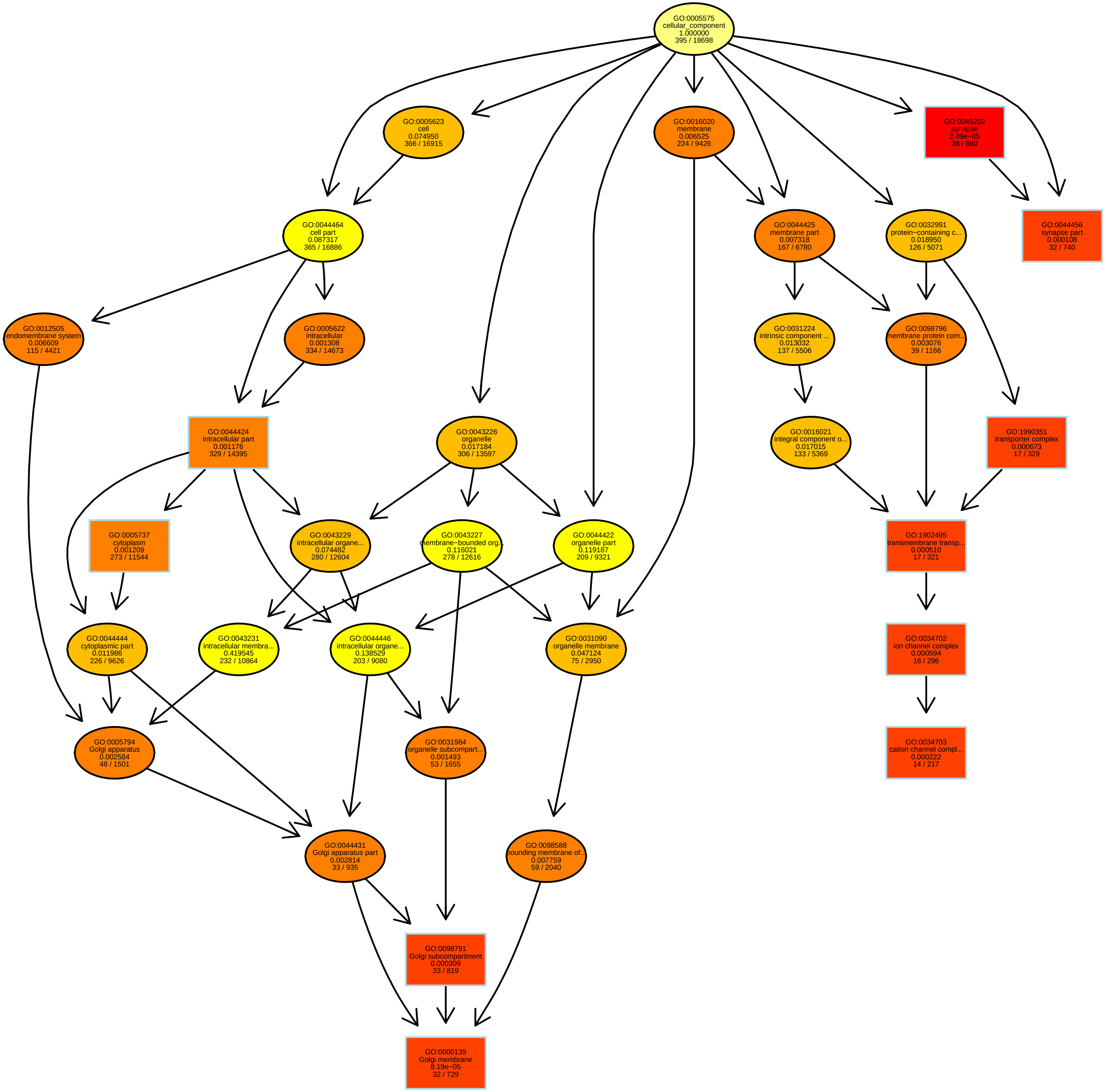
GO:0005575 cellular_component 1.000000 395 / 18698
GO:0005623 cell 0.074950 366 / 16915
GO:0016020 membrane 0.006525 224 / 9426
GO:0045202 synapse 2.49e−05 38 / 880
GO:0044464 cell part 0.087317 365 / 16886
GO:0044425 membrane part 0.007318 167 / 6780
GO:0032991 protein−containing c... 0.018950 126 / 5071
GO:0044456 synapse part 0.000108 32 / 740
GO:0012505 endomembrane system 0.006609 115 / 4421
GO:0005622 intracellular 0.001308 334 / 14673
GO:0031224 intrinsic component ... 0.013032 137 / 5506
GO:0098796 membrane protein com... 0.003076 39 / 1166
GO:0044424 intracellular part 0.001176 329 / 14395
GO:0043226 organelle 0.017184 306 / 13597
GO:0016021 integral component o... 0.017015 133 / 5369
GO:1990351 transporter complex 0.000673 17 / 329
GO:0005737 cytoplasm 0.001209 273 / 11544
GO:0043229 intracellular organe... 0.074482 280 / 12604
GO:0043227 membrane−bounded org... 0.116021 278 / 12616
GO:0044422 organelle part 0.119187 209 / 9321
GO:1902495 transmembrane transp... 0.000510 17 / 321
GO:0044444 cytoplasmic part 0.011986 226 / 9626
GO:0043231 intracellular membra... 0.419545 232 / 10864
GO:0044446 intracellular organe... 0.138529 203 / 9080
GO:0031090 organelle membrane 0.047124 75 / 2950
GO:0034702 ion channel complex 0.000594 16 / 296
GO:0005794 Golgi apparatus 0.002584 48 / 1501
GO:0031984 organelle subcompart... 0.001493 53 / 1655
GO:0034703 cation channel compl... 0.000222 14 / 217
GO:0044431 Golgi apparatus part 0.002814 33 / 935
GO:0098588 bounding membrane of... 0.007759 59 / 2040
GO:0098791 Golgi subcompartment 0.000309 33 / 819
GO:0000139 Golgi membrane 8.19e−05 32 / 729
